# TARFA: A Novel Approach to Targeted Accounting Range Factor Analysis for Asset Allocation

**DOI:** 10.3390/e28010052

**Published:** 2025-12-31

**Authors:** Jose Juan de Leon, Francesca Medda

**Affiliations:** Institute of Finance and Technology, University College London, London WC1E 6BT, UK; f.medda@ucl.ac.uk

**Keywords:** comparable analysis, comparable valuation, entropy, equity analysis, equity valuation, factor-based models, value investing, C58, C63, G11, G12, G14, G17, G32, M41

## Abstract

The valuation of companies has long been a cornerstone of financial analysis and investment decision-making, offering critical frameworks for investors to gauge a firm’s worth and evaluate the relative value of future income streams within a specific industry or sector. In this work we propose a new valuation framework by integrating traditional and modern valuation approaches, providing actionable insights for investors and analysts seeking to optimize asset allocation and portfolio performance. We introduce a novel framework (TARFA) to comparable company valuation by identifying investor-preferred return-driving points for accounting-based factors. Through an analysis of 68 commonly used accounting measures, the study identifies three key factors that drive superior returns. The results of the TARFA framework demonstrate that both general and sector-specific models consistently outperformed population returns, with the general model showing superior performance in broader market contexts. The study also highlights the stability of key financial ratios over time and introduces the Relative Equity Score, further enhancing the model’s ability to identify undervalued equities.

## 1. Introduction

The valuation of companies has long been a central concern of finance and investment, providing essential tools for estimating the worth of firms and the relative value of future income streams within and across industries [1,2]. Historically, fundamental analysis has relied on widely accepted metrics and market multiples such as price-to-earnings (P/E) ratios and enterprise value to earnings before interest, taxes, depreciation, and amortization (EV/EBITDA) as the backbone of relative valuation [3,4,5]. As Frensidy et al. [6] highlight, the P/E ratio remains the most commonly employed accrual-based valuation model for industry comparison, while discounted cash flow (DCF) methods are often preferred for deriving target prices due to their higher precision. When combined, these approaches can improve valuation accuracy and reduce dispersion in estimates.

Despite their widespread use, traditional valuation models often fail to capture the full informational content embedded in financial statements. A growing body of empirical research shows that financial statements contain a much broader set of ratios with meaningful predictive power beyond the limited set typically employed in conventional valuation practice [7,8,9]. Investors and analysts increasingly incorporate detailed accounting indicators when forming expectations and pricing assets, highlighting the importance of exploiting the full informational richness of firm fundamentals [10,11]. However, most applications analyse these ratios in isolation or in small groups, without a systematic framework to identify the specific value ranges in which they best explain returns. As a result, the potential of accounting information to inform asset pricing often remains under-utilised.

The factor-pricing literature faces similar limitations. Despite extensive advances in fundamental valuation and factor modelling, academic and practitioner research continues to show that a considerable portion of cross-sectional return variation remains unexplained by standard models [12,13,14]. Traditional accounting ratios are widely used, yet they are commonly applied in fixed functional forms and without attention to the specific value ranges in which they exert the strongest predictive power, which can lead to inefficiencies in capital allocation [7,8]. Furthermore, the explosion of accounting-derived variables identified in empirical finance has raised concerns about data-snooping and model proliferation [15]. These challenges create a practical and theoretical need to develop a systematic, transparent, and empirically grounded method to isolate the accounting ranges most relevant for asset pricing.

To address the limitations identified in both valuation and asset-pricing literature, this paper introduces Targeted Accounting Range Factor Analysis (TARFA), a valuation and asset-pricing framework that systematically identifies the accounting factor ranges most strongly associated with excess returns. While TARFA represents a methodological contribution, the motivation for its development is grounded in persistent limitations in existing approaches, which do not systematically identify the precise accounting ranges where return predictability is strongest [7,8]. TARFA addresses these issues by isolating empirically validated accounting ranges that are most strongly linked to excess returns, offering a transparent and scalable method for factor construction. The contribution is therefore twofold. First, it provides a structured mechanism to translate granular accounting information into asset-pricing factors in a reproducible manner. Second, it enhances interpretability by grounding factor formation in observable financial statement behaviour rather than ad hoc variable selection. TARFA leverages advanced econometric techniques to refine traditional metrics by focusing on the segments of accounting variables that are empirically linked to superior performance [9,16]. In doing so, TARFA extends rather than replaces traditional valuation tools, ensuring their continued relevance for modern investors [17,18].

The importance of TARFA is magnified by the growing complexity of capital markets and evolving financial reporting standards [19,20]. By pinpointing and weighting accounting ratios according to their empirical relevance, TARFA enables analysts and asset managers to integrate a much richer information set into portfolio construction. This approach links fundamental data more directly to real-world performance outcomes, complementing classical methods with precision that is grounded in market behaviour [7,10].

Empirically, TARFA is applied to an initial universe of 68 widely studied accounting-based factors, which are filtered and ranked based on their contribution to explaining excess returns. The analysis ultimately identifies three key ratios, Gross Profit to Total Assets (GP/TA), Inventory Turnover Ratio (ITR), and Dividend Payout Ratio (DPR), as the most robust drivers of return. These ratios serve as a proof of concept for the methodology. Using a robust statistical design, each factor is segmented into deciles and evaluated using the Gibbons et al. [21] Gibbons, Ross, and Shanken (GRS) framework to determine the specific ranges most strongly associated with superior performance. Portfolios constructed around these identified ranges consistently outperform the market, achieving an annualised excess return of over 39 percent in out of sample testing, compared with 7.2 percent for the general market population.

These findings build on a strong foundation in the factor-pricing literature. The five-factor model developed by Fama and French [13] underscores the importance of integrating multiple dimensions of firm performance into asset pricing. Novy-Marx [8] further demonstrates the profitability premium’s relevance, especially the role of the Gross Profit to Assets ratio. Harvey [15] also provide robust evidence on the performance of multi-factor models across different economic environments, supporting the empirical strength of TARFA’s design. In this sense, TARFA can be understood as an evolution rather than a departure. It refines the existing valuation and factor modelling toolkit by embedding empirically verified accounting ranges directly into the asset-pricing process. Rather than adding ad hoc factors, TARFA aims to systematically identify the points where investors price accounting information most strongly, providing a replicable and transparent framework for portfolio construction [22,23,24,25].

Methodologically, the paper contributes by operationalising range based accounting factors within a GRS framework and by complementing standard variance-based diagnostics with an entropy based measure of how concentrated return-predictive content is across deciles.

The remainder of this paper is structured as follows. Section 2 provides a comprehensive literature review, situating the study within both traditional valuation research and the modern accounting-based asset-pricing literature. Section 3 presents the conceptual motivation for TARFA, outlining the need for a systematic approach to identifying value-relevant accounting ranges and positioning the framework within existing empirical and theoretical debates. Section 4 introduces the proposed methodology. Section 5 details the intermediate steps and concessions embedded in the methodology. Section 6 presents the econometric results. Section 7 applies these findings to backtest a trading strategy constructed using TARFA. Section 8 concludes.

## 2. Literature Review

Traditional valuation theory positions firm value as the discounted present value of future cash flows. In practice, this foundational principle underpins models used by both practitioners and academics. However, equity markets have evolved substantially, particularly with the rise of high growth and intangible-intensive firms whose future cash flows are harder to forecast with precision. This has driven researchers to scrutinise the limits of conventional valuation techniques and explore the informational content embedded in accounting data.

Traditionally company valuation distils ultimately to worth being the present value of future cash flows, however with the introduction of high growth companies the difficulty to predict cash flows has drastically increased, leading to a search for new proxies to identify value. Recent studies have highlighted the limitations of relying solely on these traditional metrics, emphasizing that they may overlook critical accounting-based factors that significantly impact investor preferences and subsequent returns [7,26]. For instance, non-traditional factors such as earnings quality, revenue recognition practices, and discretionary accruals are now recognized as influential in shaping market perceptions and driving stock performance [16,27]. Moreover, the evolving landscape of financial reporting, driven by the adoption of International Financial Reporting Standards (IFRS) and increased regulatory scrutiny, has heightened the importance of understanding these accounting nuances within the valuation process [19,20].

In addition to issues concerning information completeness, researchers have noted challenges in comparative valuation techniques. While traditional metrics provide a straightforward means of comparing companies, they are often critiqued for their inability to capture the full spectrum of factors influencing a company’s true market value [28,29]. This critique includes not only the comparability of these ratios but also the selection process of peer groups. Alford [28] demonstrated that combining risk and earnings growth is effective for selecting comparable firms. Separately, recent research by Geertsema and Lu [30] has explored the application of machine learning models in valuation, finding that these models significantly outperform traditional ones in accuracy. Additionally, their analysis of principal components revealed important factors in stock valuation, echoing some elements of earlier findings by Liu et al. [29], who identified forward earnings measures as the most accurate, followed by historical earnings measures, with cash flow measures and book value of equity tied for third, and sales measures performing the worst.

Taken together, this body of literature establishes that conventional valuation models remain essential but incomplete. There is clear academic support for expanding the set of financial indicators used in valuation, particularly those that capture profitability, operating efficiency, and payout policy.

## 3. Conceptual Motivation and Foundations for TARFA

Although the literature has advanced beyond simple multiples and cash flow models, two persistent methodological issues remain. First, studies often analyse accounting metrics in isolation rather than as part of a structured system. Second, most existing approaches assume a uniform or monotonic relationship between accounting variables and returns. The empirical asset-pricing literature rarely identifies the specific value ranges of accounting measures that drive performance. This creates a methodological gap that motivates a more granular and systematic range-based approach.

It is clear that traditional valuation models like P/E ratios and the DCF approach have been central to investment analysis, however recent advancements in the field have emphasized the importance of integrating more nuanced, accounting-based factors into these frameworks. This research builds on these methodologies by introducing the TARFA framework, which refines valuation approaches by focusing on three key ratios: GP/TA, ITR, and DPR. These ratios were selected for their proven ability to drive returns, with Gross Profit to Total Assets highlighting profitability, Inventory Turnover Ratio reflecting operational efficiency, and Dividend Payout Ratio offering insights into shareholder value distribution. By incorporating these metrics, TARFA aims to enhance the precision and relevance of traditional models, ensuring they remain effective in today’s complex financial markets.

The focus on Gross Profit to Total Assets aligns with recent research highlighting profitability as a critical driver of firm [8,20]. Profitability has long been identified as a key determinant of firm performance, as firms with higher gross profitability are often more efficient in utilizing their assets, leading to superior returns. The strong empirical evidence supporting this metric has led to its widespread adoption in both academic research and practical applications in asset allocation [8,13,17,20].

Inventory Turnover Ratio, another key metric, is well recognized for its crucial role in efficient capital management and its significant impact on a firm’s operational performance, which directly influences stock returns. Extensive research has demonstrated this relationship in the manufacturing Kwak [31] and retail industries Hancerliogullari et al. [32], Gaur et al. [33], showing that efficient inventory management can enhance cash flows and profitability. However, the broader market impact of Inventory Turnover Ratio remains less explored, suggesting an area for further study in terms of general market conditions and its potential role in influencing broader financial outcomes.

Finally, the Dividend Payout Ratio remains a vital factor for investors, particularly in assessing a firm’s commitment to returning value to shareholders, a strategy closely associated with sustainable financial practices and often interpreted as a signal of financial health and stability [34]. The DPR has been extensively studied in the context of dividend signaling theories, such as the Future Earnings Hypothesis, which posits that dividends serve as a signal of a firm’s future profitability [35,36,37]. However, empirical support for this hypothesis has been mixed, with studies finding little evidence of a strong connection between dividend changes and future earnings [38,39,40].

In contrast, the Risk Reduction Hypothesis suggests that dividends communicate a reduction in firm risk rather than future earnings, with numerous studies finding a negative relationship between dividend yield and firm risk [41,42,43]. This effect has been notably highlighted in the banking sector, where research shows a positive relationship between lagged dividends and future financial health, indicating that dividends can be used as a tool to signal reduced risk conditions to investors [44,45]. The commitment to regular dividends, especially in highly regulated industries such as banking, thus becomes a crucial indicator of financial stability and is often adopted as a mechanism to pacify investors during times of economic uncertainty [46,47].

In summary, the literature recognises that accounting information is central to forecasting returns, yet lacks a systematic approach to identify and exploit the specific value ranges where accounting variables exhibit the strongest predictive power. TARFA responds to this gap by operationalising range-based accounting factor identification and testing across the full return distribution, thereby advancing the asset-pricing toolkit.

## 4. Methodology

The TARFA methodology proceeds through a series of structured stages designed to systematically translate raw accounting information into return-predictive factors and equity signals. The procedure begins by identifying the relevant investment universe. This initial population-identification step ensures that only appropriate firms are considered, aligning with established practices in asset-pricing research and allowing for consistency in reporting standards, liquidity, and investability. The objective at this stage is simply to form the complete cross-section of stocks against which subsequent accounting signals will be evaluated.

Once the population is defined, the set of potential accounting factors is constructed. To ensure breadth and replicability, we focus on the 68 most commonly used financial ratios identified within Wharton Research Data Services (WRDS). Each ratio is computed monthly for every stock in the universe. The cross-section of stocks is then shorted into deciles based on the value of each accounting measure, rebalancing monthly. For each decile, the corresponding average monthly return is computed. This decile-sorting procedure follows the empirical tradition in factor construction and allows us to observe how returns vary across different regions of each accounting distribution rather than assuming fixed linear effects.

With the decile portfolios constructed, the predictive relevance of each decile is evaluated. Each decile portfolio is treated as a candidate factor, and its ability to explain cross-sectional variation in returns is examined. Specifically, we compute excess returns by subtracting the risk-free rate from each decile portfolio return, and we incorporate each resulting excess-return series as an additional explanatory factor in the traditional Fama-French five-factor model. The analysis then computes the Gibbons, Ross, and Shanken (1989) F-statistic (GRS) to assess whether the augmented model improves explanatory power. To ensure comparability across factors, the scores are normalised the resulting GRS scores by the maximum value within each ratio and portfolio, averaging them to obtain a unified performance metric for each ratio-decile combination, referred to as the Average Score, bounded between zero and one.

The following stage converts the Average Score into a Decile Score for each ratio. This transformation allows us to map individual firms to the specific value regions of each accounting variable that exhibit return-relevant properties, rather than assuming uniform factor influence across the full ratio distribution. Unlike traditional factor studies that focus on entire variables, TARFA evaluates the empirical meaning of each decile separately.

Ratio selection is then performed by identifying the variables whose deciles exert the strongest and most heterogeneous impact on returns. Specifically, the GRS based performance is compared between the top and bottom deciles for each ratio. Ratios with the largest spread are retained, as they exhibit the clearest differentiation in return behavior across their range. This step mitigates factor dilution and addresses concerns of factor proliferation by emphasising only variables with demonstrable economic and statistical relevance.

Finally, once the relevant ratios and their Decile Scores are determined, we assign each stock an Equity Score. For each firm, we identify which decile it belongs to for every selected ratio and sum the corresponding Decile Scores. The resulting Equity Score reflects the aggregate return-predictive accounting signal implied by a firm’s position across the key accounting dimensions. These scores ultimately form the basis for portfolio formation and performance evaluation, linking accounting structure to investment outcomes in a transparent and empirically validated manner.

The subsequent sections expand on this framework, providing additional detail regarding design choices, implementation considerations, and robustness procedures.

### 4.1. Population Identification

Prior to conducting the data analysis, it is essential to establish a well-defined population. In our case, we select a comprehensive set of indices, particularly ensuring that all stocks within them are included if they have been traded for at least one full month. This approach helps us avoid survivorship bias by capturing all relevant stocks, as long as they have traded for one month or longer.

In our analysis, two kinds of populations are tested, both matching the population employed in Fama and French [13,48,49]. One is denominated as *general*, where the whole population is assessed, and the other as *specific*, where only particular industries are included. These specific industries are identified through their North American Industry Classification System (NAICS) code.

### 4.2. Factor Construction

After identifying all the ratios and stocks, we categorize the data into ten deciles for each ratio. This is done by ordering the equities based on the numerical values of the corresponding ratios monthly. Stock returns are calculated and assigned to their respective stocks, and the stocks within each decile are averaged. The output can be visualized as a table where each factor has ten columns, representing the average return for each decile group, with rows corresponding to the dates.

To minimize the impact of non-comparable companies, companies without a data point for the required multiple are not considered. When splitting the data into deciles, these are treated as data points, but the decile is not generated. As a result, a GRS is not obtained (Tables 1–5). This particularly allows us to capture a long-term image of investor behavior in the market and remove outliers.

### 4.3. Factor Assessment

We now assess the ability of particular deciles and ratios at driving returns. Each factor previously computed is regressed in conjunction with the original five factors as introduced in Fama and French (2015), following Equation (1). This factor is consecutively exchanged and tested by different combinations of each factor and all factors.

(1) $$\begin{array}{cc} E\left(R_{i}\right)-R_{f} & =\alpha_{i}+\beta_{i}\cdot \left(E\left(R_{m}\right)-R_{f}\right)+\beta_{j}\cdot {\mathrm{SMB}}_{t}+\beta_{k}\cdot {\mathrm{HML}}_{t}\\ \; & \;+\beta_{l}\cdot {\mathrm{RMW}}_{t}+\beta_{m}\cdot {\mathrm{CMA}}_{t}\\ \; & \;+\beta_{s}\cdot \left({\mathrm{ChangingTerm}}_{t}-\left(E\left(R_{m}\right)-R_{f}\right)\right)+e_{it} \end{array}$$

In the model, $E\left(R_{i}\right)-R_{f}$ represents the excess returns of portfolio *i* over the risk-free rate, capturing the additional gain expected from the portfolio compared to a secure investment. $E\left(R_{m}\right)-R_{f}$ designates the excess market returns, capturing the overall market’s performance above the risk-free rate. ${\mathrm{SMB}}_{t}$ represents the excess returns of small-capitalization companies over large-capitalization companies, highlighting the size effect in asset pricing. ${\mathrm{HML}}_{t}$ quantifies the excess returns of stocks with high book-to-price ratios over those with low book-to-price ratios, indicating the value premium. ${\mathrm{RMW}}_{t}$ measures the difference in returns between diversified portfolios of stocks with robust and weak profitability, while ${\mathrm{CMA}}_{t}$ captures the differential returns between portfolios of low and high investment firms. ${\mathrm{ChangingTerm}}_{t}$ is a placeholder for the introduced factors. The error term $e_{it}$ accounts for idiosyncratic risks not explained by these factors. The beta coefficients reflect the exposure to these respective factors, and the intercept $\alpha_{i}$ is used to obtain the GRS F-Value score.

Once a GRS score is obtained for every factor, decile combination, and portfolio, each GRS value is normalised by the maximum observed for that factor and portfolio. The resulting normalised scores are then averaged across portfolios. This procedure yields a single score for every decile and factor, denoted as ${\mathrm{AverageScore}}_{i,j}$, where *i* denotes the factor and *j* the decile, bounded between 0 and 1.

By employing linear regression and GRS, we test the ability of each factor to improve the regression employed. The factors are tested against the risk-free rate portfolios proposed by Fama and French. The tested portfolios may be segmented into those with value-weighted and equal-weighted returns. This is further divided by firm sizes, from small to large, and by book-to-market ratios, with the ranges from low to high being subdivided into five categories. The GRS statistic is computed for every group of five, where they are equal in size but each pertains to one of the five different book-to-market categorizations.

### 4.4. Scoring Methodology

Following the values obtained in the previous section, the deciles of the different ratios are ordered from smallest to greatest, based on the order and the score attributed to each decile for each factor. All equities across all months are scored according to Equation (2).

(2) $${\mathrm{DecileScore}}_{i,j}=\frac{1}{{\mathrm{Order}}_{j}}\times \frac{1}{\mathrm{Average}{\mathrm{Score}}_{i,j}}$$

where ${\mathrm{DecileScore}}_{i,j}$ is the score for factor *i* in decile *j*, ${\mathrm{Order}}_{j}$ is the rank from smallest to greatest of the factor in decile *j*, and ${\mathrm{AverageScore}}_{i,j}$ is the average for each factor and decile of all portfolios’ GRS scores normalized by the maximum for each factor and portfolio.

To construct and map the corresponding ratio value ranges to the deciles, two different approaches are taken. The first consists of obtaining the score for the whole population from the training part of the dataset and assuming this applies to individual sectors. The second consists of obtaining the score for the individual sectors from the training part of the dataset and applying it to the respective sector. The boundaries identified in deciles are matched to corresponding decile ranges and employed in the backtesting stage when attributing scores to individual equities. Results are presented for both cases, the first being designated as the *general* approach and the second as *specific*.

### 4.5. Ratios Selection

To identify factors that consistently contribute to explaining results, we compared the average GRS value for each decile against the GRS score obtained by using only the standard five Fama and French factors. We considered only those factors that enhanced the GRS value in 64% or more of instances, reducing our sample from 68 factors to 5. We tested for the overall dataset, identifying which factors for individual deciles in all portfolios reduced the GRS score in contrast to the traditional five-factor methodology. This was done to identify the factors with the most explanatory power improving deciles. The analysis results are displayed in Appendix A.1 Table A1.

This factor analysis may be seen as independent of the rest of the analysis as it is only directed towards determining which factors to consider. It must be noted that we do not claim these factors to be the best proxies, but only a good proxy to demonstrate that we may divide ratios into deciles, which may help understand the ranges preferred by investors. The five surviving factors are described as follows (all following the definition by WRDS):

(3) $$\mathrm{GP}/\mathrm{TA}=\frac{\mathrm{GrossProfit}}{\mathrm{TotalAssets}}$$

where GP/TA is the ratio of Gross Profitability (GrossProfit) to Total Assets (TotalAssets).

(4) $$\mathrm{ITR}=\frac{\mathrm{CostOfGoodsSold}}{\mathrm{AverageInventory}}$$

where ITR is the fraction of Cost of Goods Sold (CostOfGoodsSold) divided by Average Inventories (AverageInventory) based on the most recent two periods.

(5) $$\mathrm{DPR}=\frac{\mathrm{Dividends}}{\mathrm{NetIncome}}$$

where DPR is the fraction of Dividends (Dividends) divided by Income Before Extra Items (NetIncome).

(6) $$\mathrm{efftax}=\frac{\mathrm{IncomeTax}}{\mathrm{PreTaxIncome}}$$

where efftax is Income Tax (IncomeTax) as a fraction of Pretax Income (PreTaxIncome).

(7) $$\mathrm{adv}\_\mathrm{sale}=\frac{\mathrm{AdvertisingExpense}}{\mathrm{Sales}}$$

where adv_sale is Advertising Expenses (AdvertisingExpense) as a fraction of Sales (Sales).

Further filtering based on intrinsic characteristics led us to exclude efftax and adv_sale. In all the performed analyses, the five identified factors displayed an improvement in GRS score concerning the GRS score obtained from the traditional five Fama and French factors in more than 64% of tested instances as previously described. However, some of the factors may introduce biases in nature, as they may discriminate against stocks different in nature from those where these factors are vital.

These two characteristics being efftax, as it may introduce biases toward multinational companies with better tax management. Another factor with a similar bias is adv_sale, which was excluded as not all industries use advertising to the same degree, again indirectly attributing a higher score to companies where advertisement is central to operations. This refinement brought the number of factors down to three.

### 4.6. Equities Scoring Methodology

Once the Decile Score is obtained, every equity is attributed a monthly score based on the previously defined Decile Score per Equation (8), taking the form of the sum of the Decile Scores previously introduced in Section 4.4, for the three preserved ratios:

(8) $$\begin{array}{cc} \mathrm{Equity}{\mathrm{Score}}_{k} & =\mathrm{Decile}{\mathrm{Score}}_{\left(\mathrm{GP}/\mathrm{TA},j\right)}+\mathrm{Decile}{\mathrm{Score}}_{\left(\mathrm{ITR},j\right)}\\ \; & \;+\mathrm{Decile}{\mathrm{Score}}_{\left(\mathrm{DPR},j\right)} \end{array}$$

The financial data date is set by the public_date from WRDS. Public_date designates the month-end dates matched with adate and qdate, where adate is the end of the fiscal year for annual financial data, and qdate is the date when quarterly financial statements are compiled and reported. Public_date is rolled over to be the first date of the following month, with the opening price of the stock on this date assumed to be the date the portfolio is rebalanced. This approach prevents any lookahead bias by allowing a two-month gap from the actual date of financial data reporting to when the data is considered publicly available and usable in analysis.

The scores are generated with respect to the population of all NYSE, AMEX, and NASDAQ publicly traded equities filtered as previously described, or individual industries. Specific scores are generated individually for NAICS 21 and 22. NAICS sector 21, which encompasses Mining, Quarrying, and Oil and Gas Extraction, is referred to as “Petrol” in this paper. Meanwhile, NAICS sector 22 corresponds to Utilities. The approach where the overall population is considered is denominated general, and the industry-centric one is specific. The results are obtained and compared.

Taking the form of a continuation of the presented methodology in the next section, we evaluate the robustness of the TARFA scores using three complementary diagnostics: training-window stability, multicollinearity checks based on VIF, and an entropy-based measure that summarises how dispersed the decile-level predictive content is for each ratio.

## 5. Methodology Evaluation

The purpose of this section is to assess whether the TARFA methodology behaves in a consistent, stable, and economically meaningful manner. Although the previous section outlined the construction of the Equity Score and the underlying financial ratios, it is necessary to evaluate the decisions made during the design process and verify that the results are not driven by arbitrary modelling choices or sample-specific effects. This evaluation proceeds in several steps.

First, we examine the effect of using different training sample lengths to determine whether the decile weights and factor rankings remain stable when the model is estimated using shorter or longer historical windows. This is motivated by the need to verify that the scoring system is not overly sensitive to the sample period and instead reflects persistent return patterns in the data.

Second, we assess whether the selected ratios demonstrate reliable explanatory power relative to established asset-pricing factors. This includes evaluating multicollinearity with the Fama and French five factors and confirming that the constructed ratios contribute distinct information rather than replicating existing factor exposures.

Finally, we compare the resulting scores and return patterns across industries and time periods to establish whether the methodology captures economically intuitive relationships, both in sample and out of sample. Together, these steps provide a structured assessment of the robustness and interpretability of the TARFA framework before turning to the empirical results and backtesting analysis.

### 5.1. Effect of Training Dataset Size

To test stability, we re-estimate the model on three windows: (i) full sample, January 1997–January 2024 (325 months); (ii) pre-2020 window, January 1997–January 2017 (241 months); and (iii) growth-tilted window, January 2020–July 2022 (31 months).

Across Table 1 and Table 2 the ordering and magnitudes of DPR, ITR, and GP/TA deciles are similar, indicating stable weights when moving from 325 to 241 months of data. The growth-tilted window in Table 3 shows narrower DPR dispersion but wider ITR dispersion, consistent with a period when growth was favoured over value. Despite these shifts, the relative patterns that drive returns in Section 7 persist, suggesting the scoring remains robust to window length while capturing regime-specific preferences. As the general-case universe is larger, its Range Size is naturally wider than the sector-specific cases discussed later, which supports stronger cross-sectional differentiation in the broad model.

### 5.2. Econometric Model Multicollinearity Assessment

To examine the multicollinearity of the identified factors before performing GRS analysis, the Variance Inflation Factor (VIF) was calculated. Most factors displayed moderate correlation, with most factors’ values under 5, not warranting corrective action. All the studied factors presented values between 0.99 and 5.34. The three factors assessed in further detail are presented in Table 4. Additionally, the employed backtesting model performed well at predicting returns in the out of sample test, highlighting the robustness of the selected factors. The GRS scores and associated measures are presented in [50].

Taken together, the evidence in this section indicates that the TARFA framework behaves consistently across sample periods, market regimes, and sector contexts. The stability of the decile weights when trained on different data lengths, the absence of problematic multicollinearity with standard Fama-French factors, and the economic interpretability of the ratio–return patterns all support the internal validity of the approach. These results suggest that TARFA captures persistent information in accounting variables rather than sample-specific noise or latent exposures to existing factors. This provides a reliable foundation for the econometric analysis and portfolio backtesting that follows in the next section.

### 5.3. Entropy-Based Assessment of Decile Concentration

The diagnostics in Section 5.1 and Section 5.2 so far focus on variance-based measures (GRS statistics, VIF) and factor stability. Related work in financial machine learning also highlights the importance of ensuring model robustness and stability, particularly when predictive systems are exposed to high-dimensional or adversarial environments [51]. To complement these, we introduce an information-theoretic perspective based on entropy. In information theory, entropy measures the uncertainty or dispersion of a probability distribution [52]. When applied to finance, entropy has been used as a non-parametric measure of risk, diversification, and model uncertainty, providing an alternative or complement to variance-based criteria [53,54,55]. In the context of TARFA, entropy offers a compact way to summarise how concentrated or diffuse the return-predictive content of each accounting ratio is across its deciles.

For a discrete probability vector $p=(p_{1},\ldots ,p_{J})$, the Shannon entropy is defined as

(9) $$H\left(p\right)=-\sum_{j=1}^{J}p_{j}\log p_{j},$$

with the convention that 0log0=0. To facilitate interpretation, we use a normalised entropy

(10) $$H^{*}\left(p\right)=\frac{H(p)}{\log J}\in \left[0,1\right],$$

so that $H^{*}\left(p\right)=0$ indicates full concentration in a single state and $H^{*}\left(p\right)=1$ corresponds to a uniform distribution. In our setting, lower entropy indicates that the predictive power of a ratio is concentrated in a small number of deciles, whereas higher entropy indicates that performance is more evenly spread across deciles.

#### Factor-Level Entropy of TARFA Deciles

TARFA is designed to identify the value ranges (deciles) within each accounting ratio that carry the strongest return-predictive content. The Decile Score ${\mathrm{DecileScore}}_{i,j}$ in Equation (2) already encapsulates both the ordering of deciles and their GRS-based performance. To characterise how concentrated this predictive content is across deciles for each ratio *i*, we construct a probability distribution over deciles by normalising the Decile Scores:

(11) $$p_{i,j}=\frac{\mathrm{Decile}{\mathrm{Score}}_{i,j}}{\sum_{h=1}^{J_{i}}\mathrm{Decile}{\mathrm{Score}}_{i,h}},\;j=1,\ldots ,J_{i},$$

where $J_{i}$ is the number of available deciles for ratio *i* (e.g., $J_{i}=8$ for DPR and $J_{i}=10$ for ITR and GP/TA in the general case). The factor-level entropy for ratio *i* is then given by

(12) $$H_{i}^{*}=H^{*}\;\left(p_{i,1},\ldots ,p_{i,J_{i}}\right).$$

Using the Average Scores reported in Table 2 for the Jan. 1997–Jan. 2017 general-sample estimation window, the resulting normalised entropies are

(13) $$\begin{array}{cc} H_{\mathrm{DPR}}^{*} & \approx 0.79, \end{array}$$

(14) $$\begin{array}{cc} H_{\mathrm{ITR}}^{*} & \approx 0.85, \end{array}$$

(15) $$\begin{array}{cc} H_{\mathrm{GP}/\mathrm{TA}}^{*} & \approx 0.83. \end{array}$$

As shown in Equations (13)–(15), all three ratios display relatively high normalised entropy, indicating that their return-predictive content is not confined to a single extreme decile but instead distributed across several regions of the distribution. Among the three, DPR exhibits the lowest entropy (Equation (13)), consistent with a more sharply defined set of “preferred” payout ranges. ITR shows the highest entropy (Equation (14)), suggesting that its incremental explanatory power is more diffuse across deciles. GP/TA lies in between (Equation (15)), reflecting strong high-profitability deciles together with non-negligible contributions from intermediate ranges.

Taken together, the entropy results reinforce the earlier range-size and mapping diagnostics: TARFA identifies specific ratio regions that matter for returns, particularly for profitability and payout, while still leveraging information across multiple deciles rather than concentrating all predictive power in a narrow band. Entropy therefore provides an information-theoretic confirmation that the TARFA factors are both targeted and sufficiently diversified across the ratio distributions.

## 6. Results

For analysis purposes, two models are created: one where the Average Scores of each respective industry are employed, and another where the general Average Scores are used for both the overall market and each individual industry. The first approach reflects an investor evaluating firms within a single sector using sector-specific benchmarks, whereas the second approach mirrors cross-industry stock selection based on economy-wide financial performance patterns.

This section presents the empirical results of the econometric analysis conducted on the selected accounting ratios. We begin by comparing the decile-level Average Scores for each ratio across the general market, the utilities sector, and the petroleum sector. These scores reflect the explanatory strength of each decile in the GRS framework, where lower values indicate better factor performance.

The obtained Average Scores for the DPR, ITR, and GP/TA are reported in Table 5, Table 6 and Table 7. As will be observed, the general case consistently displays the widest dispersion in scores, suggesting greater differentiation in return-predictive power across firms when evaluated in a broad-market context. In contrast, sector-specific models exhibit compressed score ranges, consistent with tighter financial structure homogeneity within industries.

The remainder of this section analyses these patterns and evaluates whether the TARFA framework identifies stable, economically meaningful return drivers across deciles, sectors, and sample periods.

Overall, these results confirm that the general-market calibration produces a wider dispersion in scores and therefore sharper differentiation in expected return patterns, while sector-specific calibration reflects more homogeneous financial structures. In the next subsections, we examine whether these observed score patterns translate into realised return behaviour and whether the TARFA factors remain stable when evaluated through both empirical and theoretical lenses.

### 6.1. Experimental Validation

To validate the empirical behaviour of the TARFA methodology, this section examines whether the factors identified in the previous section consistently align with realised return patterns. The objective is to verify that the factors with the strongest econometric performance also correspond to superior stock returns, both in sample and out of sample. This step is necessary to confirm that the methodology is capturing return-relevant information rather than overfitting to a specific period or statistical anomaly.

We begin by analysing the general case. Table 8 and Table 9 report the relationship between Average Scores, ratio ranges, and realised returns across deciles for the in-sample period (1997 to 2017) and the out of sample period (2017 to 2024). The purpose of this comparison is to observe whether the deciles that achieve the best econometric fit are also those that deliver higher realised returns. This mapping shows that deciles with lower Average Scores tend to generate higher returns, confirming that the factors selected are persistent return drivers rather than temporary artefacts.

The results are consistent across both sample periods, indicating that the methodology identifies stable relationships in investor preferences and firm characteristics, rather than short-term sentiment effects. Importantly, the pattern also holds in ratios with relatively narrow dispersion, such as the Inventory Turnover Ratio reported in Table 9. This supports the importance of incorporating ordering effects in the computation of ${\mathrm{DecileScore}}_{i,j}$, as presented in Section 4.5.

Extending this mapping to profitability, Table 10, alongside Table 8 and Table 9, checks whether lower Average Scores for GP/TA also align with stronger realised returns across both sample windows.

### 6.2. Theoretical Validation

As previously explored, traditional valuation methodologies such as the GGM are applied in the specific industries studied. Namely, utilities and petrol, utilities being the one with the highest predictability, whilst petrol is chosen as it provides a middle case between predictability and the occurrence of occasional externalities. This choice is driven by the fact that the main assumptions made for this model tend to align with the industry characteristics. These industries often exhibit lower volatility, higher predictability in their financial performance, and the payment of stable representative dividends of operations, which makes them well-suited for valuation using models that assume stable growth rates and consistent returns.

We recognize that the GGM is prevalent in some industries, and we strive to identify not the equality but the relationships between its components. To observe these relationships, below we extend the formula delineated in the GGM by introducing a DuPont analysis-like framework, allowing us to understand investors’ rationale behind traditional comparable analysis and how introducing the investor’s point of preference as the return-driving range may generate excess market returns, yielding Equation (16), by using the ratio definitions previously delineated in Section 4.5.

(16) $$P_{0}=\frac{\mathrm{TotalAssets}\times \frac{\mathrm{GP}}{\mathrm{TA}}\times \mathrm{NIM}\times \mathrm{DPR}}{r-\mathrm{ROE}\times (1-\mathrm{DPR})}$$

Let $P_{0}$ be the current stock price, NIM the Net Income Margin as defined in Equation (17), *r* the required rate of return, and ROE the Return on Equity as defined in Equation (18). The rest of the variables follow their previous definitions.

(17) $$\mathrm{NIM}=\frac{\mathrm{Net}\mathrm{Income}}{\mathrm{Gross}\mathrm{Profit}}$$

(18) $$\mathrm{ROE}=\frac{\mathrm{Net}\mathrm{Income}}{\mathrm{Shareholder}’s\mathrm{Equity}}$$

We can associate ITR to GP/TA through Equation (19), where *k* is a proportionality constant that relates Inventory Turnover to Gross Profit to Total Assets:

(19) $$\frac{\mathrm{GP}}{\mathrm{TA}}=k\times \mathrm{ITR}$$

where *k* is a proportionality constant that relates Inventory Turnover to GP/TA, and the rest of the variables follow their previous definitions.

We can observe that the formula dictates that the intrinsic price of a stock is directly proportional to Gross Profit to Total Assets and the Inventory Turnover Ratio. However, we may not derive a clear relationship for the Dividend Payout Ratio as there is a trade-off present. It stands to reason that the higher ROE is, the closer it is to *r*, yielding a higher $P_{0}$. This situation tends to occur when DPR approaches 0, allowing for further growth. However, since DPR is also a multiplier, a balance must be reached.

We can then impose the restriction that

r>ROE×(1−DPR)

in Equation (16), as the price of a stock may not be negative due to limited liability. If we consider the case when

r=ROE−ROE×DPR

rearranging gives:

(20) $$\mathrm{DPR}=\frac{\mathrm{ROE}-r}{\mathrm{ROE}}$$

Substituting this into Equation (16), we obtain:

(21) $$P_{0}=\frac{\mathrm{TotalAssets}\times \frac{\mathrm{GP}}{\mathrm{TA}}\times \mathrm{NIM}\times \left(\frac{\mathrm{ROE}-r}{\mathrm{ROE}}\right)}{0}$$

To respect the imposed condition,

DPR≈0

as theorized.

This empirically derived relationship corresponds to the values obtained through econometric analysis, where in the general case per Table 5, Table 6 and Table 7, for GP/TA the highest ratio values drive returns. Similarly, although not as clear, the highest five deciles in ITR are better return drivers than the lowest five. Additionally, as per our hypothesis, a lower DPR drives returns.

When growth is preferred over value investing, the trends are reversed, signifying that the assumptions extrapolated from the GGM no longer apply, as observed in Section 5. Despite this preference in the market, it must be noted that our methodology outperforms the market. This may be due to the long-term trend still being underlying, despite the temporal influence being different. We now proceed to backtest the identified relationships.

## 7. Backtesting

This section evaluates whether TARFA derived signals translate into implementable excess returns. We first detail portfolio formation and rebalancing rules that are common to all tests. We then define two ranking strategies, the Equity Score and a valuation-adjusted Relative Equity Score, and report results for the full sample, the out of sample period, and an S&P 500 only universe to assess large-cap robustness.

### 7.1. Backtesting Methodology

Two long only strategies are tested, each selecting the top 30 stocks each month and holding them for one month before rebalancing. The first strategy ranks stocks by the Equity Score in Equation (8). The second strategy ranks by a normalized variant, the Relative Equity Score in Equation (22), which adjusts the Equity Score using the firm’s price-to-book (PTB) characteristic to account for cross-sectional valuation differences.

(22) $$\mathrm{Relative}\;\mathrm{Equity}\;{\mathrm{Score}}_{k}=\frac{\mathrm{Equity}\;{\mathrm{Score}}_{k}+\mathrm{PTB}\;{\mathrm{Score}}_{k}}{{\mathrm{PTB}}_{k}}$$

Here, ${\mathrm{PTB}}_{k}$ is the PTB ratio of stock *k*, defined as market value of equity divided by book value of equity. $\mathrm{PTB}\;{\mathrm{Score}}_{k}$ is the contribution of the PTB ratio under the same scoring procedure used for other accounting ratios. The goal of this normalization is to recover signals that are strong relative to contemporaneous valuations rather than in absolute terms only. Both strategies are implemented under identical rebalancing and portfolio sizing rules so that performance differences can be attributed to the scoring choice.

### 7.2. Backtesting Results

#### 7.2.1. Equity Score Backtesting Results

**Figure 1:**
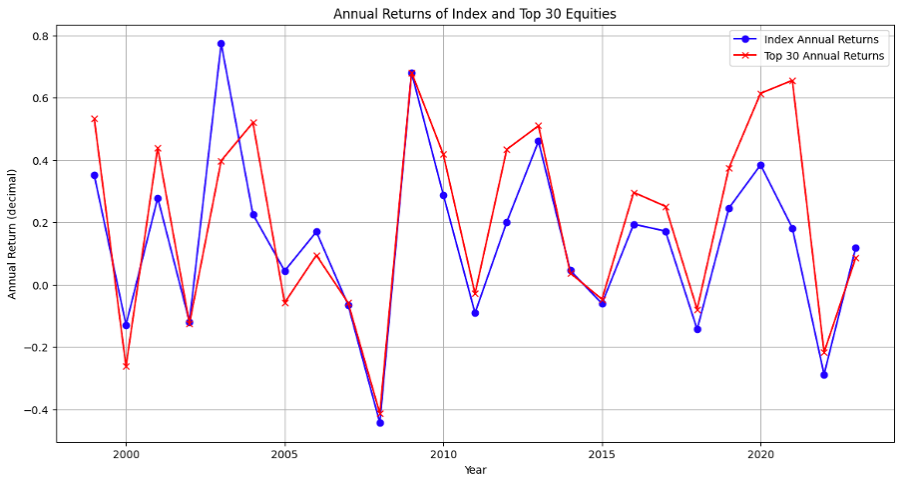
Difference of portfolio and population return vs. year for Jan. 1999 to Jan. 2024.

The model performance and its contrasting population for both the full period and out of sample test are presented in Table 11 and Table 12. The annual performance is presented in Figure 1. Overall, both general and sector specific implementations outperform their respective populations, with the general model typically delivering the strongest results; the exception in the petrol out of sample case suggests that sector breadth and sample size matter for robustness. The annual performance is presented in Table 13. The performance of commonly used indices as proxies is presented in Table 13. The proposed backtesting includes in the testing portfolio the 30 equities with the highest Equity Score, rebalanced monthly.

The results show that both the specific and general models outperform the population return in most instances, except for the out of sample period where the petrol-specific approach is inferior to the general model. Similarly, our model, both in-sample and out of sample, when selecting between all equities, outperforms all identified indexes, as shown in Table 13. This may be due to the limited number of equities in some periods for the individual sectors. Additionally, investors may not only compare equities within a sector but also across sectors. However, the results show that sometimes comparing within an industry is worthwhile.

The analysis then shifts to an index wide perspective, specifically focusing on the S&P 500 through a similar lens, employing population weights against the S&P 500-centric ones. This analysis is conducted to argue that the methodology in the general case also outperforms the market when only high-market-capitalization stocks are included. We also investigate whether performing comparisons at an index level is better than at a market level.

As shown in Table 13, the results indicate that an edge can be achieved by taking a market-wide perspective, rather than focusing on a particular subset, such as the 500 largest market capitalization stocks traded in the United States. This leads to the conclusion that comparisons should be performed across market capitalizations and not treat companies differently because of their size.

In summary, monthly portfolios formed on the Equity Score outperform sector populations and broad indices in most cases, with stronger and more stable performance when the broader market universe is available.

#### 7.2.2. Relative Equity Score Backtesting Results

To understand the impact of investor preferred deciles, we now divide by their respective PTB value the identified scores given to every equity every month to the market capitalization of the company.

It is an interesting insight that currently, the PTB is, on average, going against the hypothesis of the High minus Low Book-to-Market ratio by Fama and French. High PTB, or its inverse low PTB, seems to be driving returns, as seen in Table 14. Here we introduce our PTB score and divide it by the PTB value to understand whether the hypothesized factor—and, in turn, value investing in PTB terms—is not working. This conclusion was also recently reached by Rasmussen [56], as the value factor of Fama and French has generated negative returns in the last 14 years.

**Figure 2:**
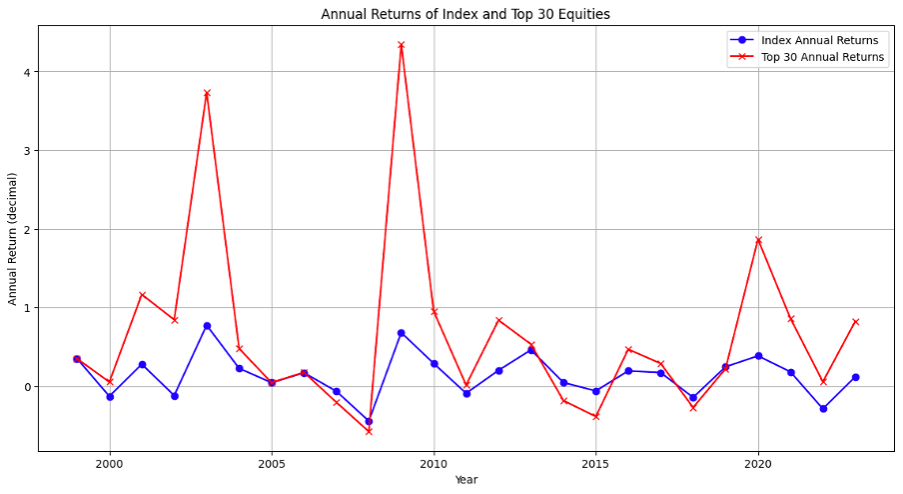
Difference in portfolio and population return vs. year for Jan. 1999 to Jan. 2024.

To incorporate this effect, we relativize the score by introducing the Relative Equity Score, as per Equation (22). The backtesting results are presented in Table 15 and Table 16. Figure 2 provides a detailed view of the yearly return of the general approach and the index. The proposed backtesting includes the 30 equities with the largest Equity Score in the testing portfolio, rebalanced monthly.

By normalizing the scores with the introduction of PTB, we could further increase returns compared to the case where the Equity Score was employed. This occurred in all instances except for utilities, where, due to their predictable nature, we were not capable of generating excess returns compared to the strategy centered on the Equity Score.

The obtained results further emphasize that a market-wide approach to investing is beneficial, as the larger number of equities available for selection allows for identifying companies that deviate significantly from market norms. By employing the Relative Equity Score, the objective is to identify high-quality, relatively undervalued companies. This further demonstrates that value investing is not a trend but an underlying principle in the market, and that companies tend to eliminate undervaluation over time. It must be noted that since only the top 30 equities are included monthly, value investing, as shown in Table 15, may on average not be profitable, as low PTB companies generate negative returns. However, as the name indicates, careful selection may generate excess market returns.

However, the proposed approach may present limitations. By introducing PTB with no constraints, it may lead to a high-risk strategy involving investments in distressed companies. This risk may be mitigated by our approach of examining financial accounting ratios. Despite this, our results show positive returns.

**Figure 3:**
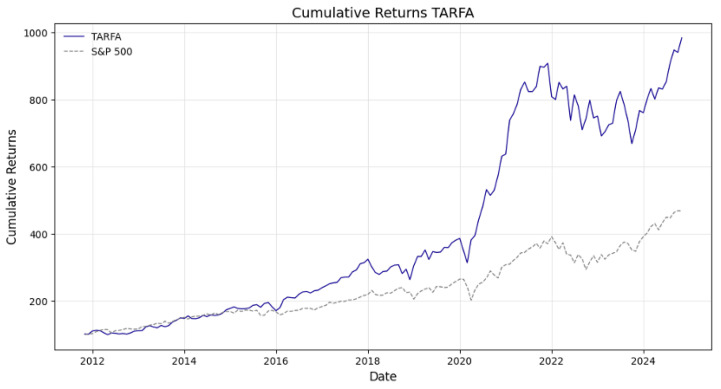
TARFA general approach and S&P 500 cumulative returns from 1 January 2011 to 31 November 2024, backtested on S&P 500 equities only.

Similarly to the previous case, the analysis then applies the methodology to an S&P 500 restricted universe. As shown in Table 17, the general approach outperforms both the market and the other approaches. There is underperformance when employing the value strategy as opposed to the previously devised strategy in Section 6, a phenomenon not previously occurring when the S&P 500-wide constraint was not applied. This emphasizes the earlier point that by choosing low PTB distressed companies, we may inadvertently invest in underperforming stocks. However, if the population is large, there are plenty of choices, and on average, we can beat the market. In this scenario, the smaller the selection pool, the worse the performance. The S&P 500-specific approach underperforms the USA-incorporated approach because the range of options is smaller in the former. Despite this, as per Figure 3 and Table 18, the approach heavily outperforms the benchmark in both average absolute returns and relative return metrics (alpha) whilst maintaining a low correlation.

The following section outlines the conclusions drawn from this paper.

## 8. Conclusions

This study set out to explore a novel approach to comparable company valuation by introducing the TARFA framework, which integrates accounting-based factors into valuation models. By analysing 68 accounting metrics, we identified three key factors, Gross Profit to Total Assets, Inventory Turnover Ratio, and Dividend Payout Ratio, that drive superior returns. Through comprehensive back testing across both full period and out of sample datasets, we validated the strength of general and sector-specific models. This section summarizes the key insights from the study, discussing their implications, limitations, and avenues for future research.

As with any empirical study, the results should be interpreted in light of the data and design choices underpinning the analysis. The empirical setting relies on U.S. equity data from Wharton Research Data Services (WRDS), covering firms listed on the NYSE, AMEX, and NASDAQ. This provides a consistent and well-established testing environment, but it also means that the findings may not fully extend to markets with different institutional structures, accounting regimes, or investor bases. Similarly, the sector-specific investigations concentrate on NAICS 21 and 22, where the cross-sectional coverage of firms varies across time. In some periods, a more limited number of observations may reduce statistical stability and constrain the ability to generalise sector-level conclusions.

From a methodological perspective, portfolio construction is based on fixed decile thresholds that are held constant over time. While this choice supports interpretability and facilitates comparisons across samples, it does not explicitly accommodate changes in market regimes or shifts in the relative importance of accounting signals. In addition, the backtesting framework abstracts from transaction costs, liquidity considerations, and short-selling frictions. As a result, the documented performance should be viewed as indicative of the underlying economic signal rather than a direct representation of net, implementable returns. Finally, although conservative reporting lags are applied to mitigate look-ahead bias, some residual timing effects associated with accounting disclosures may persist. Collectively, these considerations suggest that the findings are best interpreted as evidence of the robustness and relevance of the TARFA framework, while leaving scope for further refinement in more granular or implementation-focused settings.

The findings of this study provide important insights for refining both traditional valuation methodologies and modern investment strategies. First, the consistent outperformance of the general model across multiple market contexts underscores the value of adopting a broad market-wide perspective. This approach, combined with the Relative Equity Score, allowed for the identification of high-quality, undervalued stocks, which significantly outperformed sector-specific models, particularly in the broader market.

Sector-specific models still proved useful in certain industries, particularly in those with well-established return-driving factors, such as petrol and utilities. However, the superior performance of the general model across sectors and the broader market, including indices such as the S&P 500, suggests that investors benefit more from looking beyond sector or index boundaries. For example, in the out of sample period, the general model outperformed the S&P 500 index (16.91% versus 11.61%), further reinforcing the advantage of a market-wide approach.

While this study provides robust findings, there are some limitations to consider. First, the performance of the sector-specific models, particularly in sectors like petrol, was sometimes limited by the small number of available equities in certain periods, restricting the ability to capture broader market trends. Additionally, while the general model performed well across most market conditions, in highly stable sectors like utilities, sector-specific models performed similarly, limiting the relative advantage of a broader market perspective.

Additionally, the internal validity of TARFA is supported by entropy-based diagnostics, which show that the predictive content of the key ratios is concentrated yet not restricted to a single extreme decile, complementing the variance-based robustness checks performed earlier in the analysis.

This study opens up several potential avenues for future research. One promising area would be to apply the TARFA framework to markets outside of the U.S., particularly in emerging economies where financial market dynamics may differ significantly. Further exploration of sectors with less defined return-driving factors, such as technology and energy, may also reveal valuable insights into the broader applicability of the TARFA model in more volatile or less mature industries.

Future research could also refine the Relative Equity Score by incorporating additional financial metrics beyond the PTB, such as earnings yield or cash flow metrics. These could provide a more comprehensive view of company valuation, particularly in sectors where traditional valuation factors may have limited predictive power. Additionally, exploring the cyclical nature of value versus growth investing, especially in response to macroeconomic shifts, could provide valuable insights into how investor preferences evolve over time and how these changes impact long-term investment strategies.

In conclusion, this study makes a meaningful contribution to the field of comparable company valuation by introducing a refined framework that integrates traditional accounting-based factors with advanced econometric techniques. The general model, combined with the Relative Equity Score, consistently outperforms both sector-specific models and population returns, especially in broader market contexts. This research reinforces the value of adopting a market-wide perspective in modern investment strategies and provides a powerful tool for identifying return driving stocks. Despite some limitations, the TARFA framework demonstrates strong potential for improving both traditional and contemporary investment strategies, reaffirming the continued relevance of accounting-based factors in today’s financial markets.

While TARFA demonstrates the enduring value of accounting-based factors and provides a systematic approach to factor construction across industries, it also highlights the boundaries of fundamentals alone. Financial statements capture the quantitative side of firm performance, but they cannot account for the qualitative dimension of how markets interpret and react to information. Recent evidence shows that linguistic structure in earnings calls can generate return-predictive signals beyond traditional factors, reinforcing the need to integrate both quantitative and qualitative information in valuation frameworks [57]. Investor sentiment, as shaped by managerial language in earnings calls and corporate communications, plays a central role in driving valuations—particularly in innovation-driven sectors where expectations often outweigh historical performance.

Overall, the backtesting evidence confirms that TARFA can translate accounting information into systematic and persistent return premia. In practice, this implies that TARFA may serve as a complementary tool within fundamental investing workflows, providing a structured and data driven mechanism to rank firms by accounting quality and expected performance. The approach therefore offers a practical contribution to equity selection, portfolio construction, and factor investing research by operationalising comparable company analysis in a transparent and repeatable manner.

## Figures and Tables

**Table 1:** Average Scores, full sample (Jan 1997–Jan 2024; 325 months).

Decile	DPR	ITR	GP/TA
1	0.294	0.379	0.403
2	0.559	0.322	0.446
3	0.696	0.333	0.400
4	0.737	0.342	0.370
5	0.727	0.335	0.342
6	0.772	0.326	0.309
7	0.698	0.315	0.283
8	0.695	0.318	0.268
9	-	0.328	0.255
10	-	0.389	0.265
Range Size	0.478	0.074	0.191

Note: “Range Size” is the difference between the largest and smallest Average Score in each ratio column; it is not a sample size.

**Table 2:** Average Scores, pre-2020 window (Jan 1997–Jan 2017; 241 months).

Decile	DPR	ITR	GP/TA
1	0.336	0.353	0.348
2	0.468	0.348	0.396
3	0.537	0.360	0.381
4	0.532	0.371	0.349
5	0.529	0.355	0.360
6	0.567	0.344	0.340
7	0.519	0.336	0.302
8	0.638	0.359	0.292
9	-	0.395	0.275
10	-	0.378	0.296
Range Size	0.302	0.059	0.121

Note: “Range Size” is the difference between the largest and smallest Average Score in each ratio column; it is not a sample size.

**Table 3:** Average Scores, growth-tilted window (Jan 2020–Jul 2022; 31 months).

Decile	DPR	ITR	GP/TA
1	0.578	0.557	0.399
2	0.460	0.527	0.520
3	0.472	0.556	0.513
4	0.475	0.561	0.371
5	0.453	0.473	0.396
6	0.502	0.475	0.408
7	0.463	0.473	0.459
8	0.498	0.470	0.474
9	-	0.424	0.463
10	-	0.323	0.454
Range Size	0.125	0.238	0.149

Note: “Range Size” is the difference between the largest and smallest Average Score in each ratio column; it is not a sample size.

**Table 4:** Variance inflation factors vs. Fama–French five factors (Jan 1997–Jan 2024).

Decile	DPR	ITR	GP/TA
1	3.35	2.95	2.68
2	4.19	3.17	2.99
3	4.82	3.33	3.40
4	5.12	3.42	3.08
5	5.16	3.41	3.19
6	5.34	3.39	3.35
7	4.92	3.16	3.38
8	4.66	3.31	3.23
9	-	3.36	2.95
10	-	2.96	2.50

Note. Values rounded to nearest integer.

**Table 5:** Dividend Payout Ratio Average Score.

Decile	Petrol	Utilities	General
1	0.396	0.415	0.336
2	0.407	0.431	0.468
3	0.399	0.437	0.537
4	0.397	0.434	0.532
5	0.400	0.441	0.529
6	0.393	0.434	0.567
7	0.386	0.434	0.519
8	0.382	0.438	0.638
9	-	0.435	-
10	-	0.422	-
Range Size	0.025	0.026	0.302

Note. Lower values indicate better regression fit; Decile 1 = lowest ratio values, 10 = highest.

**Table 6:** Inventory Turnover Ratio Average Score.

Decile	Petrol	Utilities	General
1	0.398	0.411	0.353
2	0.393	0.421	0.348
3	0.393	0.420	0.360
4	0.406	0.417	0.371
5	0.398	0.426	0.355
6	0.396	0.427	0.344
7	0.395	0.422	0.336
8	0.397	0.431	0.359
9	0.400	0.431	0.395
10	0.395	0.425	0.378
Range Size	0.013	0.020	0.059

Note. Lower values indicate better regression fit; Decile 1 = lowest ratio values, 10 = highest.

**Table 7:** Gross Profit to Total Assets Ratio Average Score.

Decile	Petrol	Utilities	General
1	0.390	0.405	0.348
2	0.391	0.408	0.396
3	0.393	0.423	0.381
4	0.395	0.420	0.349
5	0.396	0.423	0.360
6	0.396	0.425	0.340
7	0.397	0.423	0.302
8	0.395	0.437	0.292
9	0.394	0.428	0.275
10	0.392	0.429	0.296
Range Size	0.007	0.032	0.121

Note. Lower values indicate better regression fit; Decile 1 = lowest ratio values, 10 = highest.

**Table 8:** Scores, range, and returns for Dividend Payout Ratio.

Decile	Avg.	DPR Range		Return	
	Score	Min	Max	1997–2017	2017–2024
1	**0.336**	−1363.9	0.087	0.081	0.031
2	**0.468**	0.087	0.167	0.091	0.040
3	0.537	0.167	0.244	0.080	0.020
4	0.532	0.244	0.328	0.075	0.014
5	0.529	0.328	0.429	0.070	0.008
6	0.567	0.429	0.577	0.070	0.001
7	0.519	0.577	0.896	0.069	0.001
8	0.638	0.896	22,913.6	0.076	0.006
9	-	-	-	-	-
10	-	-	-	-	-

Note. Annualised returns in base 1; two smallest Average Scores shown in bold.

**Table 9:** Scores, range, and returns for Inventory Turnover Ratio.

Decile	Avg.	ITR Range		Return	
	Score	Min	Max	1997–2017	2017–2024
1	**0.353**	−61.363	1.588	0.043	−0.029
2	**0.348**	1.588	2.496	0.057	−0.015
3	0.360	2.496	3.329	0.064	−0.002
4	0.371	3.329	4.247	0.067	0.000
5	0.355	4.247	5.388	0.070	0.004
6	0.344	5.388	7.2	0.068	0.004
7	0.336	7.2	10.7538	0.067	0.003
8	0.359	10.7538	19.6152	0.059	−0.003
9	0.395	19.6152	51.085	0.059	−0.005
10	0.378	51.085	137,211	0.058	−0.013

Note. Annualised returns in base 1; two smallest Average Scores shown in bold.

**Table 10:** Scores, range, and returns for Gross Profit to Total Assets.

Decile	Avg.	GP/TA Range		Return	
	Score	Min	Max	1997–2017	2017–2024
1	**0.348**	−134.24	0.031	0.008	−0.068
2	0.396	0.031	0.050	0.033	−0.030
3	0.381	0.050	0.115	0.048	−0.002
4	**0.349**	0.115	0.194	0.058	0.008
5	0.360	0.194	0.262	0.072	0.020
6	0.340	0.262	0.330	0.083	0.031
7	0.302	0.330	0.407	0.090	0.035
8	0.292	0.407	0.504	0.098	0.049
9	0.275	0.504	0.669	0.098	0.050
10	0.296	0.669	8.680	0.094	0.047

Note. Annualised returns in base 1; two smallest Average Scores shown in bold.

**Table 11:** Full period model performance. All returns are adjusted for dividends and splits.

Period (Jan. 1999–Jan. 2024)	Average Yearly Return		
	Utilities	Petrol	All Industries
	NAICS 22	NAICS 21	All NAICS
Population (only relevant sector)	10.31%	8.89%	10.47%
Model general	11.26%	11.23%	15.87%
Model specific	10.98%	13.30%	-

**Table 12:** Out of sample model performance. All returns are adjusted for dividends and splits.

Period	Average Yearly Return		
Out of Sample Test	Utilities	Petrol	All Industries
(Jan. 2017–Jan. 2024)	NAICS 22	NAICS 21	All NAICS
Population (only relevant sector)	9.22%	1.78%	7.19%
Model general	12.60%	7.87%	20.07%
Model specific	13.02%	10.1%	-

**Table 13:** Index performance with reinvested dividends.

Index	Average Yearly Return	
	Reinvested Dividends	
	Jan. 2017–Jan. 2024	Jan. 1999–Jan. 2024
Russell 2000	7.34%	7.91%
Russell 1000	13.22%	7.72%
Russell 3000	12.82%	7.74%
S&P 500	11.61%	6.86%

**Table 14:** Scores, range, and return mapping for the Price-to-Book factor. The annualized return is given in base 1. The two smallest Average Score deciles and their related characteristics are presented in bold.

Decile	Avg. Score	PTB Min	PTB Max	Return	
				1997–2017	2017–2024
1	0.294	0.056	0.661	−0.004	−0.083
2	0.336	0.661	0.936	0.028	−0.046
3	0.349	0.936	1.173	0.042	−0.028
4	0.362	1.173	1.428	0.052	−0.016
5	0.315	1.428	1.732	0.061	−0.003
6	0.284	1.732	2.127	0.071	0.013
7	0.249	2.127	2.692	0.081	0.024
8	0.225	2.692	3.637	0.087	0.038
**9**	**0.209**	**3.637**	**5.870**	**0.099**	**0.052**
**10**	**0.202**	**5.870**	**114.149**	**0.112**	**0.075**

**Table 15:** Full period model performance average yearly return, by employing Relative Equity Score. All returns are adjusted for dividends and splits.

Period	Utilities	Petrol	All NAICS
(Jan. 1999–Jan. 2024)	(NAICS 22)	(NAICS 21)	(All Sectors)
Population (only relevant sector)	10.31%	8.89%	10.47%
Model general	12.63%	14.71%	39.62%
Model specific	12.66%	14.60%	-

**Table 16:** Out of sample model performance average yearly return. All returns are adjusted for dividends and splits.

Period	Utilities	Petrol	All NAICS
Out of Sample Test	(NAICS 22)	(NAICS 21)	(All Sectors)
Population (only relevant sector)	9.22%	1.78%	7.19%
Model general	14.10%	12.91%	42.21%
Model specific	11.72%	11.55%	-

**Table 17:** Index performance with reinvested dividends: average yearly return under a value investing strategy.

Index	Jan. 2017–Jan. 2024 Return	Jan. 1999–Jan. 2024 Return
S&P 500 specific approach	13.87%	10.44%
S&P 500 specific approach– USA Incorporated	8.88%	1.53%
General approach	16.91%	10.61%
S&P 500	11.61%	6.86%

**Table 18:** TARFA general approach cumulative returns with respect to S&P 500 population 1 Januray 2011 to 31 November 2021 with the S&P 500 as a benchmark.

Metrics	TARFA	S&P 500
Annualised Return	0.215	0.1360
Annualised Volatility	0.1846	0.1408
Sharpe Ratio	0.97	0.7227
Sortino Ratio	0.98	1.1674
Maximum Drawdown	−0.2635	−0.2477
Downside Volatility	0.1076	0.0892
Beta	1.24	–
Average Alpha	0.082	–

## Data Availability

The empirical analysis in this study is based on proprietary data obtained from the WRDS platform, including security-level returns and accounting information for NYSE, AMEX, and NASDAQ stocks, as well as Fama–French factor and inputs employed to compute portfolio returns. Due to WRDS licensing restrictions, these datasets cannot be shared publicly. Researchers with access to WRDS can reproduce the results by querying the same datasets and variables. No additional proprietary datasets were created for this study.

## Abbreviations

The following abbreviations are used in this manuscript:

TARFA	Targeted Accounting Range Factor Analysis
WRDS	Directory of open access journals
DPR	Dividend Payout Ratio
ITR	Inventory Turnover Ratio
GP/TA	Gross Profit to Total Assets

## Appendix A. Appendix

### Appendix A.1. Percentage of Deciles Enhanced for Every Factor

**Table A1:** Percentage of deciles enhanced for every factor, for the whole dataset. The greater the percentage, the more frequently the factor yields enhancement.

Factor	%	Factor	%	Factor	%
inv_turn	82	curr_ratio	40	accrual	20
adv_sale	74	umd_market	40	opmbd	20
efftax	72	pretret_noa	38	opmad	20
GProf	66	evm	38	sale_equity	20
dpr	64	totdebt_invcap	36	roa	20
rect_act	62	cash_lt	36	pe_op_basic	18
lt_debt	58	debt_capital	34	pe_exi	16
gpm	56	short_debt	32	pe_inc	16
rect_turn	56	bm	32	int_debt	14
capital_ratio	54	sale_nwc	30	ptpm	12
dltt_be	52	quick_ratio	30	aftret_invcapx	10
equity_invcap	50	CAPEI	30	cfm	10
de_ratio	50	cash_ratio	30	aftret_eq	10
curr_debt	50	PEG_trailing	28	roe	10
pay_turn	50	cash_debt	28	sale_invcap	10
debt_assets	48	pretret_earnat	26	roce	10
debt_invcap	46	profit_lct	26	int_totdebt	10
debt_ebitda	46	ps	24	npm	10
invt_act	46	pcf	24	aftret_equity	10
debt_at	44	pe_op_dil	24		
cash_conversion	44	intcov_ratio	24		
fcf_ocf	42	ocf_lct	22		
lt_ppent	40	at_turn	22		
ptb	40	intcov	22		

### Appendix A.2. Ratio Ranges for NAICS 21 Dataset (Jan 1999–Jan 2017)

**Table A2:** Ratio ranges by decile for DPR, ITR, and GP/TA for the NAICS 21 dataset (Jan 1999–Jan 2017).

Decile	DPR		ITR		GP/TA	
	Min	Max	Min	Max	Min	Max
1	−1.266	0.043	0.284	4.657	−31.587	−0.035
2	0.043	0.071	4.657	7.473	−0.035	0.062
3	0.071	0.107	7.473	9.795	0.062	0.116
4	0.107	0.156	9.795	12.537	0.116	0.153
5	0.156	0.225	12.537	16.074	0.153	0.182
6	0.225	0.339	16.074	20.8658	0.182	0.209
7	0.339	0.616	20.865	29.464	0.209	0.238
8	0.616	65.395	29.464	42.723	0.238	0.278
9	—	—	42.723	86.044	0.278	0.343
10	—	—	86.044	52,644	0.343	0.978

### Appendix A.3. Atio Ranges Across All NAICS Sectors (Jan 1999–Jan 2017)

**Table A3:** Ratio ranges by decile for DPR, ITR, and GP/TA across all NAICS sectors (Jan 1999–Jan 2017).

Decile	DPR		ITR		GP/TA	
	Min	Max	Min	Max	Min	Max
1	−1363.9	0.087	−61.363	1.588	−134.24	0.031
2	0.087	0.167	1.588	2.496	0.031	0.050
3	0.167	0.244	2.496	3.329	0.050	0.115
4	0.244	0.328	3.329	4.247	0.115	0.194
5	0.328	0.429	4.247	5.388	0.194	0.262
6	0.429	0.577	5.388	7.200	0.262	0.330
7	0.577	0.896	7.200	10.7538	0.330	0.407
8	0.896	22,913.6	10.753	19.6152	0.407	0.504
9	—	—	19.615	51.085	0.504	0.669
10	—	—	51.085	137,211	0.669	8.680

### Appendix A.4. Ratio Ranges for NAICS 22 Dataset (Jan 1999–Jan 2017)

**Table A4:** Ratio ranges by decile for DPR, ITR, and GP/TA for the NAICS 22 dataset (Jan 1999–Jan 2017).

Decile	DPR		ITR		GP/TA	
	Min	Max	Min	Max	Min	Max
1	−6.858	0.348	0.421	7.041	−1.812	0.061
2	0.348	0.491	7.041	8.750	0.061	0.075
3	0.491	0.573	8.750	10.4844	0.075	0.083
4	0.573	0.642	10.4844	12.3912	0.083	0.089
5	0.642	0.711	12.3912	15.002	0.089	0.095
6	0.711	0.804	15.002	18.0532	0.095	0.101
7	0.804	0.977	18.0532	22.983	0.101	0.109
8	0.977	111.830	22.983	29.087	0.109	0.119
9	—	—	29.087	43.747	0.119	0.134
10	—	—	43.747	19,418.5	0.134	0.671
